# Chinese nurses' self‐expression media image during COVID‐19 pandemic: A qualitative media image analysis

**DOI:** 10.1002/nop2.1156

**Published:** 2022-01-14

**Authors:** Huili Cao, Yangjie Chen, Xingyue He, Yejun Song, Qiaohong Wang, Hui Yang

**Affiliations:** ^1^ Nursing College of Shanxi Medical University Taiyuan China; ^2^ Linfen Hospital Affiliated to Shanxi Medical University (Linfen people's Hospital) Linfen China; ^3^ The First Hospital of Shanxi Medical University Taiyuan China

**Keywords:** COVID‐19 pandemic, Nurses' self‐expression, Nurses self‐image, Qualitative media analysis

## Abstract

**Aim:**

To explore and describe nurses' self‐expression media image during COVID‐19 pandemic in China.

**Background:**

Nurses play an important role in COVID‐19 pandemic. Although nurses were widely reported by the media, which included praise for nurses and nursing work, the researches on how nurses expressed their self‐images were limited.

**Design:**

Qualitative media analysis.

**Methods:**

Qualitative media analysis was conducted from January to April 2020, the researchers collected images and texts of 16 Chinese nurses who take care of COVID‐19 patients. These images and texts were published on WeChat Moments by themselves. After analysed each image and text, researchers identified the denotative and connotative elements in each image and summarized each image in narrative way.

**Findings:**

This study analysed 219 pictures and 15 short videos of 16 nurses' self‐expression in WeChat moments. In this study, the media image self‐expression of nurses were mostly positive. The images expressed by nurses in this study included care image; hero image; soldier image; female image; hope image and team image. Nurses rarely showed negative images in the media; The negative nurses image were expressed in hidden way, which included exhausted nurses image and fragile nurses image. Moreover, the nurse self‐expression media image emphasized the nursing professionalism, but less showed the nursing connotation.

**Conclusions:**

The positive media image self‐expression of nurses should be encouraged. Nurse Managers should pay attention to the deficiency of nursing image expression and guide nurses to show the essence and connotation of nursing.

**Implications for nursing**

The nurse media image self‐expression is a necessary component of the nurse public image. Nursing managers should encourage nurses to establish positive media image on social media and show the complexity and importance of nurses' work. These measures may become windows for the public to understand nurses in public health emergencies and may enhance the public's confidence in the medical system's response to COVID‐19 pandemic.

Our study follows Standards for Reporting Qualitative Research (SRQR).

## 1 INTRODUCTION

The COVID‐19 pandemic is a great challenge to human health worldwide (World Health Organization, 2020). Countless nurses were involved in the battle of COVID‐19 pandemic. When faced with infectious diseases, nurses were regarded as heroes and professionals who had nursing virtue (Hall et al.2003; Morin & Baptiste, 2020). One study showed nurses' perception of their public profession image may improve the quality of nursing work life (Roshangar et al., 2021). Professional image is a social representation established by a set of concepts and refers us to occupational identity (da Silva et al., 2002). The public image (PI) of nursing, which incorporates beliefs, ideas and impressions that people have of nurses and nursing, has always been a socio‐cultural issue for this profession (Emeghebo, 2012; Wallace, 2007).

The nurse public image is important. Caring, attentive, empathetic, efficient, knowledgeable, competent and approachable are considered as attributes of professional, nurses own or lack them may contribute positively or negatively to the patient experience (West et al., 2016). However, the nurse public image were not always comprehensive. For example, nurses as a "necessary sacrifice" during COVID‐19 pandemic (Mohammed et al., 2021). The nurse's task is described by the nurse's caring role. When nurses work in a multi‐disciplinary team, nursing decisions were considered limited (Glerean et al., 2019). Some studies pointed out negative public image may had the direct effect on nurses' sense of identity (Almutairi et al., 2015), even affect the professional concept of some nursing students (Iersel et al., 2016). Previous studies showed that nursing was viewed as a less desirable profession due to the difficult working conditions, inadequate financial compensation, low level of autonomy, limited career opportunities and being viewed as ancillary members of the healthcare team (Racic et al., 2019). Therefore, facing COVID‐19 pandemic, we should let the public know the real nurses and nursing. The self‐expression of nurses in the media may become a window for the public to understand nurses and nursing.

Globally, nurses are encouraged to use social networking sites for online communication. More than 1.8 billion people use global social networking sites (Baker & Algorta, 2016). In China, WeChat is an important media platform for communication and expression. Social media has become an important platform for nurses to show people's profession and life. The image of nurses will affect their recruitment, retention, professional value, and ultimately affect the health and well‐being of individuals, families and communities (Girvin et al., 2016; Kelly, 2010; Nigel Crisp et al., 2018). It is worth mentioning that the affordability and accessibility of social media may break the hierarchy of discourse power (Howard et al.2011; Nicogossian, 2012; Schwab, 2016). Some researchers pointed out that nurses public image always different from the real image of nurses (Hoeve et al., 2014). In fact, nurse image is a multidimensional, all inclusive, paradoxical, dynamic and complex concept (Rezaei‐Adaryani et al., 2012).

Most cognition and image of nurses come from the media, medical experience and people’ traditional concepts, but rarely from nurses themselves. The expression of nurses' self‐image reflects the image that nurses expect the public to understand. Therefore, we focus on the self‐expression media image of nurses, which can help people deep their understanding of nursing. Nurses as health leaders also can make voices on social media to express the power of nursing. Therefore, this study used qualitative research and image analysis to explore (1) What are the nurses' images, which self‐expression during the COVID‐19 pandemic? and how them expressed themselves? (2) What are the reasons for promoting or inhibiting nurses' self‐expression during the COVID‐19 pandemic?

## 2 METHODS

### 2.1 Design

The methodological framework used in this study was the Qualitative Media Analysis (QMA) (Altheide, 2013; Morse, 1996). QMA was mainly used to analyse texts and images. We first recruited qualified nurses through the purpose sampling and snowball method. The nurses recorded their experience during COVID‐19 pandemic by pictures or texts and published on WeChat Moments. We obtained the permission to collect, download and analyse these pictures and texts. Then researchers analysed the pictures and wrote the analysis texts. By encoding the pictures and related texts, the researchers summarized the self‐expression of nurses' media image to help public understand what are the self‐expression nurse's image during COVID‐19 pandemic.

### 2.2 Data collection

The data were collected between January and April 2020. The researchers began to recruit qualified nurses to participate in the study through objective sampling. The researchers continued to find participants by snowballing.

The inclusion criteria were Registered Nurses in China; on a voluntary basis to participate in the study and agree to provide relevant pictures and texts of nurses image expression published on WeChat Moments during the COVID‐19 and nurses who care for COVID‐19 patients.

The exclusion criteria were nurses, students or non‐Registered Nurses; refuse to provide relevant pictures and texts; did not participate in COVID‐19 patients care; no pictures and words related to nurses' media image were published on social media.

Then researchers got permission to collect, download and analysis pictures of nurses self‐expression in their WeChat Moments. The images and texts were selected by the research team, which explicitly reflected the images of nurses when they care for COVID‐19 patients. When the data of the 16th participants were collected, there were no new theme appeared, the data were saturated.

### 2.3 Data analysis

We used NVivo12 pro for data analysis. Researchers focused on the pictures and tests published by WeChat Moments from health care provided during the COVID‐19 pandemic. The inclusion criteria were (1) clear pictures (not included patients’ characteristics) (2) the relevant pictures and texts of nurse's image expression published on WeChat Moments during the COVID‐19 pandemic. Then evaluators interpret the pictures write text materials to describe the connotative and denotative elements of the images, respectively (See table 1 and table 2). Finally, other members of the research group discussed the different opinions on nurses' self‐expression media image. After that, the researchers encoded the interpretation text of the picture.

**TABLE 1:** The connotative elements list of image

(1) What does the image show?
(2) What are the components of the image?
(3) Which place attracts the audience's attention? Why?
(4) What knowledge is there in the image?
(5) What knowledge is excluded from the image?

**TABLE 2:** The denotative elements list of image

(1) Who is the original audience of the image?
(2) Where is the text? How to show?
(3) Who is the nearest audience?
(4) Does the image belong to a series? What's the special significance?
(5) Does the audience interact with the image

Detailed steps (Altheide, 2013; Morse, 1996):

1. Extensive reading, sorting and searching through materials.
2. Write the interpretation text of the picture according to the list. With this narrative documentation of the visual, the researcher now expands on the image through inferences, interpretations and implications of the image through analytic memoing.
3. Comparing in categories, coding, and adding keywords and concepts.
4. Writing mini‐summaries of categories.
5. Write memos.
6. Obtain research results.

### 2.4 Rigour

The quality of this study had been strictly controlled. Evaluators systematically learned the qualitative research knowledge and were familiar with the methods of Qualitative Media Analysis.

Before data collection, the researcher established good relationship with the nurses who provided pictures and texts. The researchers adopted the strategy proposed by Koch to maintain the credibility of the data (Koch, 1994). In this study, double coding was used, and pictures and texts were analysed after discussion in the research group. Throughout the data collection and analysis process, researchers wrote memos to document any potential biases and prejudices. At the end of the study, the results were returned to the participants for comments and corrections. Researchers corrected incorrect or inconsistent expressions in time. Standards for Reporting Qualitative Research (SRQR) was used to guide this study (O'Brien et al., 2014).

### 2.5 Ethics

Our study was approved by the ethical committee of The First Hospital of Shanxi Medical University, China (approval no. 2020K061).

First, the researchers explained the study purposes to the participants in detail. Only researchers can observe and analyse these photos and texts and the data only be used for research.

Secondly, we ensured the security of photos. The researchers ensured texts confidentiality by using the code to blur the identifiable information (such as nurses N1, N2 and patients P1, P2) and deleted the identification information from the records. Researchers blurred some patients in pictures by special technology (although most of the photos of nurses' self‐expression on social media had been approved by patients and can be used for scientific research, teaching and media).

Third, all data were saved on a password‐protected computer. Additionally, following the principle of confidentiality, which can be consulted only by researchers of the study group. All data would be kept for three years after the end of the study and then destroyed.

Although the photos and texts about nurses' COVID‐19 care were displayed by themselves at WeChat Moments. However, when the researchers explained that data was used for research purposes, some nurses refused. Finally, 16 nurses agreed to participate in the study.

## 3 FINDINGS

We recruited 16 nurses from five hospital in Shanxi, China. As the nurses supporting Wuhan in China, they witnessed the whole process about COVID‐19 nursing (see table 3). Participants' age ranged from 29 to 60 years old. Two of them were male. Before arriving for Wuhan, the participants worked as traditionally nurse in Emergency Department, Intensive Care Unit, Digestive Department, etc. Some of them are nursing managers or director of nursing department. Most of the pictures were taken by nurses using mobile phones. Some details of life and work were recorded. This study analysed 219 pictures and 15 short videos of nurses' self‐expression in WeChat Moments.

**TABLE 3:** The demographical characteristics of the participants

Characteristics	Participants	
		*n*
Age, in years: M (range)	(26–60)	‐
Highest level of education	Postsecondary—diploma/certificate	3
	University graduates	5
	Master's diploma/certificate/candidate	6
	PhD candidate	2
Marital status	Married	10
	Unmarried	4
	Unclear	2
Occupation	Clinical nurse	8
	Nurse manager	6
	Director of nursing	2

### 3.1 The media image self‐expression of 16 nurses were mostly positive. There were six media images of nurses' self‐expression

After the analysis by researchers and the discussion of the study group, the media image self‐expression of nurses mostly were positive. The positively images expressed by nurses in this study included care image; hero image; soldier image; female image; hope image and team image.

#### 3.1.1 Care image

Nurses were special occupational people who provided nursing and care. In our study, nurses showed caring, comforting and helpful image. Some pictures show nurses taking care of patients beside the bed: for example, one nurse who wearing protective clothing wiping patient’ faces; One photo shows a patient receiving an infusion. She has a blood pressure cuff on her arm. She was thirsty and the nurse in protective clothing fed her water. In one picture, a nurse wrote a birthday card for COVID‐19 patient and held a birthday party for her. Nurses also try to meet some requirements of patients, such as some patients want to eat apples and pickles. The nurses provided their food to the patients. These behaviours showed the nature of nursing: caring.

#### 3.1.2 Hero image

Sixteen nurses in the study rarely thought of themselves as heroes. But the interaction between nurses and society reflected the hero image of nurses. For example, a photo showed before going to Wuhan, some nurse leaders organized activities to express their gratitude to nurses and praise them for their bravery. And one photos showed when these nurses returned, colleagues, polices and even people they didn't know greeted them back. The slogans everywhere showed gratitude to the doctors and nurses, calling them heroes. Some pictures showed the back of nurses in protective clothing, and the "reverse walker" image expressed the metaphor that nurses are like heroes. Nurses did not escape from danger but went to the most dangerous places to help people.

#### 3.1.3 Soldiers image

Nurses and soldiers are different professions. In this study, nurses have the image of soldiers, because they are the protector of people's health. The enemies for nurses are COVID‐19. Some pictures in this study showed the soldiers image of nurse through ceremonial activities. For example, before going to Wuhan, nurses swore at the National flag. For the safety of themselves and patients, they trained like soldiers repeatedly. This study showed soldiers image as taking picture with flag, using a temperature gun, and wearing protective suits like a warrior's suit. Deep indentations on nurse cheek like the scars or bullet marks left by soldiers on the battlefield. It is a symbol by taking care of COVID‐19 patients. Some pictures showed nurses had strong, perseverance, mission spirit liked soldiers in face of COVID‐19. For example, in a picture a nurse leader showed an application to the journalist. In the paper, there were 17 nurses requested the organization to agree to go to Wuhan Hospital for working. The 17 red fingerprints on the application paper showed their determination and brave. In another picture, a male nurse's clothes were drenched after finishing his work. He felt fight like a soldier.

#### 3.1.4 Female image

The nursing profession always has been labelled with femininity (Seago et al., 2006). Caring, which was the core concept of nursing, was related to femininity (Rafael, 1996). In this study, the female image of nurses were shown as follows. First, nurses were like mothers who gave selfless care to patients. In a photo, a nurse in protective clothing holding a baby to comfort her. Because the child's parents were infected with COVID‐19, the nurse had become the baby's nurse mother. Second, nurses are also the mother of their children. Some nurses take care of patients who infected COVID‐19. In the study a nurse who is a mother wrote her children's name on the protective suits. Courage and faith form the children would help the mother overcome the difficulties. Third, long hair is a characteristic of female. Some pictures showed that in order to prevent infection, many nurses cut their long hair. Cutting off long hair symbolized that nurses gave up something for occupation. Although the number of male nurses has increased year by year, female nurses still has an absolute advantage. Nurses provided intensive care for COVID‐19 patients. This is a "Women's Wall" to protect human health.

#### 3.1.5 Hope image

Nurses not only create hope for the patients and the country in the COVID‐19 pandemic but also look forward hope coming. When patients were in closed isolation ward, they had to face the fear of getting sick and lack companionship. Generally, nurses viewed their responsibilities as national roles and were content in performing these roles during the outbreak. The role of a nurse is to “hold hope” and “bridge the gap” for a family with COVID‐19 patients. Some photos in this study showed nurses taken pictures with patients who defeated COVID‐19. In the photos, nurses holding flowers and smiling, showed they brought rehabilitation and hope to patients. In our study, many nurses showed their winning gesture in the photos, which seemed to show their confidence in fighting the virus. Some pictures were nurses took pictures with cakes, sunshine and green trees, showed nurses hoped COVID‐19 virus would disappear and they can return to normal life.

#### 3.1.6 Team image

Some of the pictures in this study showed the nurses teams image. Many pictures show the group photos of nurses before they go to the epidemic area. Nurses, doctors and technicians wore uniform overalls and held up slogans that said "China will win". Wuhan will win. "which show the strength of the team?”

Some pictures show nurses working as a team, working together to overcome difficulties. For example, a nurse in the isolation ward, wearing a mask and protective clothing, wrote the information that needs to be communicated on paper through the window to communicate with the nurses outside the isolation ward. A picture shows that nurses and doctors are discussing around patients and working as a team for the rehabilitation of patients. Team image is not only reflected in the uniform clothing, unified action, but also in the work of cooperation, team's responsibility.

### 3.2 The negative nurses image were expressed in hidden way

There were rarely negative nurses image in our study. Although the nurses self‐expression image showed their positive image and professionalism. It also hided some negative image. The negative nurses images in this study were as follows:

#### 3.2.1 Exhausted nurses image

Some pictures showed the exhausted nurses image. For example one picture showed a nurse just finished her work. There were impression on her forehead, which long time wearing a mask and the indentation of the mask. There were sweat on her forehead. Her face was pale and looked tired. This was a glimpse of the nurse work. One picture showed some nurses falling asleep on the floor after too long time work. These pictures also reflected the physical challenges faced by nurses in caring for COVID‐19 patients. For example, nurses had to work in protective clothing. Some nurses felt dizziness or nausea and had to stop working. One picture showed a nurse wear a plastic face screen, which made by themselves. There were some pictures showed the tiredness nurses. For example, 4 a.m. on the shuttle bus with few peoples, after the end of caring for COVID‐19 patients, nurses sleeping tired in the bus.

#### 3.2.2 Fragile nurses image

In this study, some pictures showed the fragile nurses image. One picture showed nurses were going to other places for COVID‐19 nursing. These nurses left tears when they said goodbye to their families. The nurses produced fragile emotions in the face of the unknown disease and the missing the company for their families. A nurse cried in a video interview for her six‐year‐old daughter lack of mother's company. The text showed that when homesick, it was a period of psychological vulnerability.

### 3.3 Nurse self‐expression media image emphasized the nursing professionalism, but less showed the nursing connotation

Our research found nurses image facing challenges in this study were still inaccurate, and the complexity of nursing work had not been reflected. In this study, nursing challenges mainly focused on the work under the protective suits and working in different places. In this study, the image of nursing complexity had not been described by the analysis of pictures and texts. Most pictures took place in the ward where the nurses took care of COVID‐19 patients. For example, some pictures showed nurses provided nursing for patients, helped patients keep clean. Some pictures showed nurses cleared with the medical waste. Some pictures showed nurses taking throat swabs for patients at risk of viral infection. These pictures showed nurses professionalism were dedicated, caring, brave and altruistic. But studies had shown the nurses role in the COVID‐19 pandemic was limited. There were some pictures or texts talked about nurses helped patients to relieve the danger, but this kind of self‐expression was rare. This will not help to change the public's misunderstanding, which nurses' lack of professional complexity.

## 4 DISCUSSION

### 4.1 Our findings revealed the self‐expression image of 16 nurses was not divorced from the traditional nurses image and public expectation of nursing. But it had not yet expressed the complexity and connotation of nursing

During the period of Nightingale, nurses were described as self‐sacrificing, devotional, altruistic, anonymous and silent (Gordon & Nelson, 2005), and there were similar to the same media image expression of nurses during the COVID‐19 period. In this study, the media image self‐expression of nurses were care image; hero image; soldier image; female image; hope image and team image. Similar studies pointed out the nurses media image during the period of pandemic influenza were positive. For example, when severe acute respiratory syndrome(SARS) happened in Toronto, Canada, the virtue of nursing was highly praised. Nurses were known as heroes and professionals (Hall et al.2003). The nurse is the nearest person to the patients and patients got nurses’ help before any other healthcare experts (Buheji & Buhaid, 2020).

However, some researchers analysed nursing papers and the results showed that nursing was considered troubled profession and the understanding of nurses role was limited (Girvin et al., 2016). Nurses created a part of professional image, due to nurses’ invisibility or lack of public discourse, the actual nursing public image is diverse and uncoordinated (Ten Hoeve et al., 2014). Constructive public pictures promote the professionally recognized notions like the angels of mercy, and words used in the definition of an ordinary nurse, for instance, caring and loving. However, poor nursing images, frequently generated by the media instead of based on facts, produce negative repercussions. Nursing is often depicted to be a lesser and deficient venture to be considered as a job (Rosa et al., 2020). For example, little attention was paid to nurses and their knowledge and expertise as they worked through the Ebola event. Media coverage of major health events can demonstrate system inadequacies, but inaccurate and misleading portrayals of nurses and the nursing profession can undermine and diminish the image of the nursing profession (McGillis Hall & Kashin, 2016).

Moreover, in this study the nurse self‐expression media image emphasized the nursing professionalism but less showed the nursing connotation. For example,nurses defend patients and promote the available channels of communication with doctors and various healthcare professionals. Nurses who offered to work during the pandemic protected the rights of their patients and discussed their needs and wishes. Nurses to provide excellent physical and mental care for the sick. Experienced nurses provided patients and their families with clinical, educational and psychosocial help in every instance (Wallace et al., 2020).

Making nurses get little communal respect compared to doctors and other healthcare occupational groups (Zarea et al., 2009). Nurse leaders need to implement strategies for nurses and surrounding community to increase visibility of nurses’ roles during crisis through media and other accessible way (Abuhammad et al., 2021). The study imply that nurses' self‐expression can help the public reduce professional bias.

### 4.2 The reasons for promoting nurses' self‐expression

In our study, the expression of 16 nurses' work and life was very little in their daily life. However, during the care of the patients with COVID‐19 pandemic, a large number of relevant photos and words were published on WeChat Moments. The possible explanation was that it is a precious experience for nurses to take care of patients who infected COVID‐19 in their career. Furthermore, self‐expression showed nurses' professional identity. The special experience promotes the nurses record their feelings and experience. The image expression of nurses can be regarded as the process of self‐understanding, occupation and role. The work‐related pictures and text expressions published by nurses on WeChat Moments belong to a kind of self‐disclosure, which reflects nurses’ understanding of work.

Some researchers pointed out when nurses face the public stereotyping of nursing may affect the development of their self‐concept, collective self‐esteem and job satisfaction, and their job performance (Takase et al., 2002). Some researchers pointed out all nurses realized the damage that media describes did to the nurse image (Dame, 2009). The nurses media image may have an impact on professional recruitment; policy support for nurse service; acceptance of nursing services; and the nurses self‐image (Ben Natan & Becker, 2010; Kalisch et al., 2007). When a nurse felt nursing as a vocation or a calling, it will produce professional identity and become a key driver in their choice of career. (Eley et al., 2012). Moreover hospitals, families and friends who want to know about their work in the front line, which was a reason to promote nurses self‐expression. The progress of science and technology made it convenient to record and release, and also urged nurses to record their feelings and workings in time.

### 4.3 The reasons for inhibiting nurses' self‐expression

The media promotion reflected the expectation of the role of nurses. If nurses self‐expression were negative, it will be incompatible with this mainstream. Therefore, managers should pay attention to the nurses negative images self‐expression, although these negative images were hidden and hard to find. The reasons for less negative expression may be as follows: (1)Discourse power limited the expression of negative information; (2) Participants with negative expression were not included in the study; (3) Special experience evoked professionalism and ignored the expression of negative information; (4) Family and friends' worried about work situation; (5) Fear of negative expression might cause public panic. A study found nursing students were cautious about their Facebook posts. The survey found that for security reasons, 40% of students hesitated "sometimes" before sharing information on Facebook (Bacaksiz et al., 2020). It was suggested that nursing managers should pay attention to the nurses mental health who take care of COVID‐19 patients, whether there was negative expression or not, and do a good job of management communication. This means more guidance need from organizations and leaders.

Although nurses negative images were hidden in this paper, it still suggested nurses managers should explore reasonable ways to ease nurses' emotions. During the COVID‐19 pandemic, negative words and images may cause panic. Nursing managers should communicate well, pay attention to nurses' emotions, and give organizational support.

### 4.4 Significance of nurses' self‐image expression

During the COVID‐19 pandemic, nursing leaders and managers were in the forefront of responding to the nurse’ needs in their workplaces. All clinical environments and other clinical institutions must be took action to enhance the nurse image among themselves and community members (Abuhammad et al., 2021).

Self‐expression nurses may don't realize it, but like waves, they interact with their surroundings. Nurses' self‐expression can not only promote nurses' understanding of nursing role, but also help the public to better understand nurses and their work. Nurses should try to use social media to show the real nursing to the public, and convey the nursing professionalism (Hoeve et al., 2014).

In the future, the media may appears more and more parts of young people's lives. Z‐generation youngsters will be more familiar with media applications (Shatto & Erwin, 2016), more and more young nurses use media records to express their work life. The convenience and accessibility of public media make it convenient for nurses to use we media to express their life and work. At the same time, it also breaks the monopoly of mainstream media on nursing image expression.

In the new era, every individual can make a voice. Therefore, nurses should be encouraged to express scientific ideas and professional healthcare role (Kalisch & Kalisch, 1983). A study said if nurse students do not model their individual's online professional image, then it may be created by others (Wissinger & Stiegler, 2019), so do nurses.

## 5 IMPLICATIONS AND RECOMMENDATIONS FOR NURSING

The global Nursing Now! Campaign launched in February 2018 called on nurses all over the world to take action to change the nursing profession including the nurses image (Salvage & Stilwell, 2018). Nurses' self‐image expression is a way of professional identity, especially during the period of COVID‐19 pandemic. Positive self‐expression may important steps for nurses to walk out of people professional bias area. Health department or hospitals should encourage nurses to express themselves, so as to promote public awareness of nursing and change inappropriate cognitive bias.

### 5.1 Limitations

The following limitations of this study must be considered: (1) The media image expression of nurses obtained by objective sampling method in this study may had limitations and could not represent the whole country; (2) There may be bias in the study, individual expression may exaggerate the positive part because of the particularity of discourse power. There is no actual interview to clarify, and further study need to interview nurses and to explore the relationship between media expression and social interaction.

## 6 CONCLUSION

This study revealed the nurses images by themselves provided in WeChat Moments during their took care of COVID‐19 patient in Wuhan, China. Positive nurse images included care image; hero image; soldier image; female image; hope image and team image. Negative nurses image also hided in this study were exhausted nurses image and fragile nurses image. The positive media image self‐expression of nurses should be encouraged. Nurse managers should pay attention to the deficiency of nursing image expression and guide nurses to show the essence and connotation of nursing.

## CONFLICTS OF INTEREST

There are no conflicts of interest in this study.

## AUTHOR CONTRIBUTIONS

Huili Cao, Hui Yang, Qiaohong Wang: Made substantial contributions to conception and design; Huili Cao, Yangjie Chen, Xingyue He, Yejun Song: Acquisition of data or analysis and interpretation of data; Huili Cao, Hui Yang, Qiaohong Wang: Involved in drafting the manuscript or revising it critically for important intellectual content; Huili Cao, Hui Yang, Qiaohong Wang, Yangjie Chen, Xingyue He, Yejun Song: Given final approval for the version to be published. Each author should have participated sufficiently in the work to take public responsibility for appropriate portions of the content and agreed to be accountable for all aspects of the work in ensuring that questions related to the accuracy or integrity of any part of the work are appropriately investigated.

## ETHICAL CONSIDERATIONS

Our study was approved by the ethical committee of The First Hospital of Shanxi Medical University (approval no. 2020K061).

## Data Availability

Data are available on request due to privacy/ethical restrictions.
